# The cognitive neuroscience of ketamine in major depression

**DOI:** 10.1093/brain/awaf242

**Published:** 2025-06-30

**Authors:** Sara Costi, Chloe Wigg, Erdem Pulcu, Susannah E Murphy, Catherine J Harmer

**Affiliations:** Department of Psychiatry, University of Oxford, Oxford OX3 7JX, UK; Oxford Health Foundation Trust, Warneford Hospital, Oxford OX3 7JX, UK; Depression and Anxiety Center for Discovery and Treatment, Department of Psychiatry, Icahn School of Medicine at Mount Sinai, New York, NY 10029 USA; Department of Psychiatry, University of Oxford, Oxford OX3 7JX, UK; Oxford Health Foundation Trust, Warneford Hospital, Oxford OX3 7JX, UK; Department of Psychiatry, University of Oxford, Oxford OX3 7JX, UK; Oxford Health Foundation Trust, Warneford Hospital, Oxford OX3 7JX, UK; Department of Psychiatry, University of Oxford, Oxford OX3 7JX, UK; Oxford Health Foundation Trust, Warneford Hospital, Oxford OX3 7JX, UK; Department of Psychiatry, University of Oxford, Oxford OX3 7JX, UK; Oxford Health Foundation Trust, Warneford Hospital, Oxford OX3 7JX, UK

**Keywords:** ketamine, depression, fast-acting antidepressant, neurocognitive

## Abstract

Ketamine’s potential as a rapid-acting antidepressant was first identified in 2000, despite its long-standing use as an anaesthetic agent. Clinically, ketamine alleviates depressive symptoms, including the difficult-to-treat symptom of anhedonia, within hours, with the effects of a single dose lasting for days. Since then, research has focused on uncovering the mechanisms underlying its rapid antidepressant effects in both humans and animal models. While its molecular and cellular effects have been extensively characterized, its impact on cognitive and neuropsychological mechanisms—potential mediators of its clinical efficacy—remains an area of ongoing investigation. Preclinical studies suggest that ketamine rapidly influences the lateral habenula (involved in punishment processing) and fronto-striatal (reward) systems, reverses negative affective biases in established memories, and promotes long-term stress resilience. Translating these findings to human models is crucial, and emerging evidence suggests that ketamine engages similar mechanisms in healthy volunteer and patient groups. However, its clinical application is constrained by acute side effects and an unknown long-term safety profile. Further research into ketamine’s mechanisms of action will be essential to inform the development of novel, safer and more accessible rapid-acting antidepressants.

## Teaser

Most medications for depression require weeks to become effective. Ketamine, however, works much more rapidly, reducing symptoms within hours. Costi *et al.* explore the ways in which ketamine affects how we experience and remember emotional information, and how these mechanisms may explain its fast-acting antidepressant effects.

## Introduction

This year marks 25 years since the first randomized clinical trial tested the antidepressant effect of a sub-anaesthetic dose of ketamine in major depressive disorder (MDD). This discovery represented a major paradigm shift in the field; for the first time a rapid acting antidepressant effect was seen within hours of administration, and was sustained over subsequent days. This was in striking contrast to the clinical effects of available and approved antidepressant medications for MDD, which take weeks to elicit an antidepressant effect compared to placebo. At that time, ketamine was not a novel drug. Ketamine, a non-competitive *N*-methyl-D-aspartate (NMDA) receptor antagonist, was developed as a dissociative anaesthetic in the 1960s and was extensively used during the Vietnam War due to its favourable safety profile.^1,2^ Ketamine also became a substance of abuse and is classified as a Schedule III non-narcotic substance under the Controlled Substances Act in the USA, a Schedule 2 controlled drug under the Misuse of Drugs Regulations 2001, and a Class B controlled substance under the Misuse of Drugs Act 1971 in the UK.

**Figure 1:**
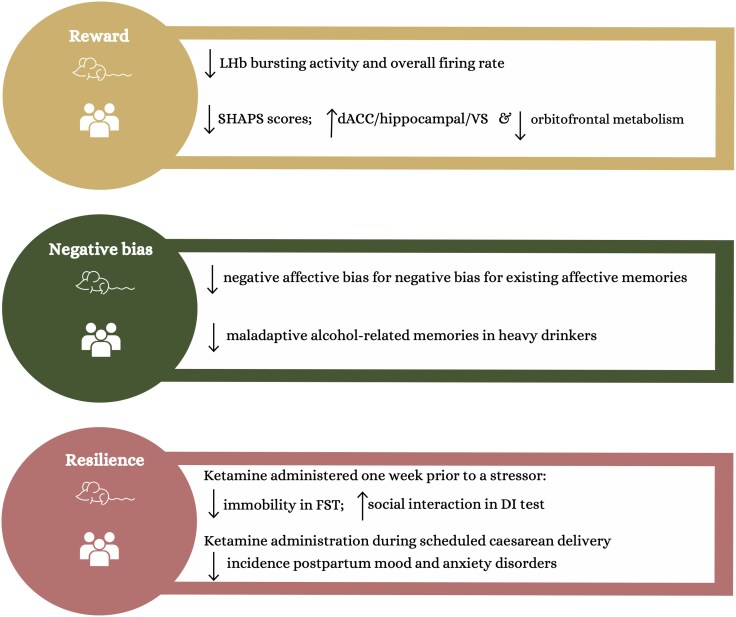
**A schematic overview of evidence of ketamine neuropsychological mechanism of action.** The figure provides a schematic representation of the neuropsychological mechanisms of ketamine’s action on reward,^4-7^ negative bias^8,9^ and resilience,^10,11^ as discussed in the current review. dACC = dorsal anterior cingulate cortex; DI = dominant interaction; FST = forced swim test; LHb = lateral habenula; SHAPS = Snaith and Hamilton Pleasure Scale; VS = ventral striatum.

Although significant advancements have been made since the first trial of ketamine’s antidepressant effects,^3^ many questions remain unanswered. As we approach this milestone, it seems timely to reflect on where we have come from and where we are heading. This review begins by presenting the clinical evidence supporting ketamine’s antidepressant effects, followed by a summary of what is currently known about its neurocognitive effects. It emphasizes ketamine modulation of the lateral habenula (LHb)—a key brain region involved in processing aversive stimuli—and its distinctive ability to modulate brain reward circuits. Finally, the review summarizes ketamine’s modulation of negative biases in established memories and discusses recent evidence of its pro-resilience effect. A schematic overview of evidence supporting its mechanism of action is presented in Fig. 1.

## A breakthrough treatment for major depressive disorder

The initial study investigating ketamine’s use in depression was designed to explore glutamate synaptic alterations in the context of MDD.^3^ This study built on preclinical findings linking NMDA receptor antagonism to antidepressant effects^12,13^ and prior research in psychosis.^14^ Ketamine was administered intravenously at a dose of 0.5 mg/kg over 40 min at a constant rate in a placebo-controlled crossover study with a small sample of medication-free MDD subjects (*n* = 7). The study demonstrated a significant reduction in depressive symptoms, measured by the Hamilton Depression Rating Scale (HAM-D), which was observed as early as 240 min post-administration and persisted for up to 72 h. Notably, the rapid and sustained antidepressant effects of ketamine were temporally distinct from its brief, acute dissociative effects.^3^ Despite the novelty of these findings, it took another 6 years before they were replicated. Zarate *et al*.^15^ reported significant improvements in depressive symptoms at 24 h and 1 week following ketamine infusion, compared to placebo, in a sample of 17 treatment-resistant depression (TRD) patients. A larger two-site trial with 73 TRD subjects also showed a similar rapid improvement in depression severity 24 h after ketamine administration.^16^ Across these trials, a single ketamine infusion in MDD was well tolerated, with response rates (defined as a ≥50% reduction in depression severity scores from baseline) ranging from 50%^3^ to 71%.^15^ In particular, ketamine treatment has been associated with improvements in anhedonia, a reduced ability to experience pleasure, which is a core symptom of depression that has proven challenging to treat with conventional antidepressants.^17^

A subsequent trial of repeated ketamine administrations [intravenous (i.v.) infusions of ketamine 0.5 mg/kg administered open-label up to three times weekly over 12 days] in MDD showed that the median time to relapse following the last ketamine infusion was 18 days.^18,19^ A study conducted in the UK testing the safety and efficacy of repeated ketamine infusions, yielded similar results.^20^ Following these initial findings, ketamine clinics have proliferated worldwide, particularly in North America, offering ketamine treatment for various mental health conditions and symptoms, including anhedonia. This evidence also led to the development of an intranasal formulation of esketamine (Spravato), which received approval by the Food and Drug Administration (FDA) and European Medicines Agency (EMA) (Box 1), the first approval of an antidepressant with a novel mechanism in 60 years. Of note, a recent meta-analysis^25^ including 87 trials on esketamine’s efficacy for depression and suicidality found that, as an add-on to antidepressants, esketamine shows modest efficacy in TRD at Weeks 2–4 (comparable to augmentation with atypical antipsychotics) but no effect on suicidality. The long-term effects of esketamine, and ketamine, remain largely unknown (Box 2).

Box 1 Key clinical trials paving the way for esketamine approval The TRANSFORM and SUSTAIN studies evaluated the efficacy and safety of esketamine nasal spray (Spravato) for treatment-resistant depression (TRD) and led to esketamine nasal spray FDA and EMA approval. TRANSFORM-2^21^ (*n* = 223) demonstrated a significant reduction in MADRS scores at Day 28, with adverse events like dissociation and nausea resolving within 1.5 h. TRANSFORM-3^22^ focused on older adults (≥65 years, *n* = 138), showing mixed results; the 65–74 age group responded better than those ≥75 years. Long-term open-label follow-up indicated sustained improvement for most patients. SUSTAIN-1^23^ involved 705 adults and evaluated the time to relapse after maintenance treatment. Esketamine reduced the risk of relapse by 51% in patients achieving remission and by 70% in those with stable response. Common adverse events included dysgeusia and somnolence. SUSTAIN-2^24^ was a long-term (up to 1 year) open-label study (*n* = 802) showing continued efficacy, with cognitive performance remaining stable. Adverse events like dizziness and nausea were mild, resolving within 1.5 h post-dose. Overall, esketamine showed sustained efficacy with manageable side effects.

Box 2 What do we know about the safety profile of ketamine for MDD? Many questions remain unanswered regarding the safety of ketamine treatment, both in acute and long-term settings. In the acute setting, ketamine’s dissociative effects make it unsuitable for use in psychotic depression, and caution is advised in the treatment of bipolar disorder due to the potential risk of inducing a manic switch. However, this risk appears to be low, as shown in a double-blind, randomized, crossover study of 15 patients with bipolar I or II depression maintained on stable doses of lithium or valproate, where a single ketamine infusion led to rapid and significant reductions in depressive symptoms and suicidal ideation within 40 min—lasting up to 3 days—without any evidence of mood switching.^26^ Additionally, whether dissociative effects are necessary for ketamine’s antidepressant effects remains a topic of considerable debate.^27-29^ In the long term, ketamine has potential for abuse, and the impact of its toxicity on the urinary bladder and brain in humans remains uncertain. The absence of long-term phase III clinical trials, combined with evidence from several trials with relatively small samples sizes, calls for caution. The most robust evidence to date comes from phase III trials of esketamine (Spravato), which suggest that long-term use is generally safe.^23,24^ The largest study on i.v. ketamine to date was a non-inferiority trial comparing ketamine with electroconvulsive therapy (ECT) for treatment-resistant depression (TRD), which included a 6-month follow-up period. This multi-site trial randomized 403 subjects (*n* = 200 ketamine) and demonstrated that ketamine was non-inferior to ECT as a therapy for TRD, with an overall safe profile.^30^

## Ketamine mechanism of action at the synaptic level

Ketamine is a non-competitive NMDA receptor antagonist that blocks the ion permeation pore located inside the NMDAR. Preclinical and clinical data suggest that this feature of ketamine is critical in generating the intracellular signalling and synaptic changes that produce antidepressant action. Central to this theory is the effect of ketamine on synapto-plasticity and dendritic spine growth in the brain, and specifically in the prefrontal cortex (PFC). Synaptic plasticity is a fundamental brain function that enables the processing and storage of information, adaptation to stimuli, and the formation of both short- and long-term memories of events.^31,32^ MDD is characterized by a reduction in synapse number, atrophy and loss of neurons and glia in brain regions such as the PFC and the hippocampus.^33,34^ In animal models, ketamine restores synaptic plasticity and reverts the atrophy that follows chronic stress.^35^

Although the mechanisms of action underlying ketamine’s antidepressant effect at the pharmacological level are not fully characterized, the two most established theories can be summarized as (i) ketamine NMDAR inhibition; and (ii) ketamine disinhibition theory (inhibition of GABAergic interneurons). Notably, these mechanisms of ketamine action are not mutually exclusive and may work together to produce the drug’s antidepressant effects.

According to the inhibition theory, ketamine selectively blocks extra-synaptic NMDARs, which are regulated by astrocytes and tonically activated by low levels of glutamate. The inhibition of these extra-synaptic NMDARs de-suppresses mammalian target of rapamycin complex 1 (mTORC1) function, subsequently inducing protein synthesis.^36^ Additionally, ketamine appears to inhibit NMDAR-mediated spontaneous neurotransmission, leading to the inhibition of eukaryotic elongation factor 2 kinase (eEF2 K) activity and preventing the phosphorylation of its substrate, eEF2. This effect enhances brain-derived neurotrophic factor (BDNF) translation.^37^ The disinhibition hypothesis suggests that ketamine selectively blocks NMDARs expressed on GABAergic inhibitory interneurons, leading to the disinhibition of pyramidal neurons and increased glutamatergic firing. The evoked release of glutamate binds to and activates post-synaptic α-amino-3-hydroxy-5-methyl-4-isoxazolepropionic acid receptors (AMPARs), resulting in increased BDNF release, activation of the tropomyosin receptor kinase B (TrkB) receptor, and subsequent promotion of protein synthesis via the activation of mTORC1.^38^ This ultimately leads to an increase in dendritic spines and synaptic plasticity.

It should be noted that other signalling pathways may be involved in ketamine antidepressant action. Of these, particular attention has been given to the opioid system. It is well established that ketamine interacts with opioid receptors, consistent with its routine use in the management of acute and chronic pain.^39^ Highlighting the potential importance of this interaction in the antidepressant action of ketamine, oral naltrexone (50 mg), an opioid receptor antagonist, administered prior to i.v. ketamine has been shown to attenuate the acute antidepressant effects of ketamine in 14 patients with depression.^40^ These findings were recently replicated in a randomized, double-blind crossover study in 26 subjects with MDD, in which naltrexone significantly attenuated ketamine’s antidepressant effects, as measured by Montgomery-Asberg Depression Rating Scale (MADRS) scores, and glutamatergic activity in the anterior cingulate cortex (ACC).^41^ However, due to the small sample size and the inability to determine whether ketamine exerts a direct or indirect effect on the opioid system, these findings should be interpreted with caution.

Finally, the effects of ketamine at the synaptic level have important translational implications for neurocognitive symptoms. For example, ketamine can induce acute, transient disruptions in cognition and perception, potentially due to a transient glutamatergic surge from disinhibition of cortical neurons via NMDAR blockade on interneurons.^42,43^ By contrast, accumulating evidence suggests that ketamine has pro-cognitive effects over the longer term in MDD, possibly through mechanisms involving AMPA receptor facilitation, mTOR signalling and synaptic plasticity.^33,44-47^

## A cognitive neuroscience perspective on ketamine’s effects

Recent advancements have deepened our understanding of how antidepressants impact neurobiological pathways and psychological functioning. While conventional antidepressants are well characterized pharmacologically, neurobiological models alone cannot fully explain the delayed mood improvements observed with treatment.^48^ Cognitive neuropsychological models have the potential to offer a useful integrative mechanistic understanding of MDD’s pathophysiology and antidepressant action.^49^ MDD is characterized by negatively biased attitudes about the self, world and future, perpetuating a cycle of negative cognitive schemas and biased processing.^50-52^ Experimental studies have shown that conventional antidepressants can modulate emotional processing early in treatment, improving the recognition of positive emotional stimuli and reducing negative biases, and that these changes are associated with later therapeutic response.^53-55^ However, what mediates the rapid acting effects of treatments, such as ketamine, is less well estbalished. The section below summarizes emerging evidence animal models and, where available from human studies, suggesting that ketamine may exhibit distinct neurocognitive effects, which may contribute to its rapid-acting profile.

### The role of the lateral habenula

Recent preclinical work has highlighted that effect on the LHb may be an important mechanism of ketamine’s antidepressant action. The LHb is an evolutionarily conserved brain region densely populated with glutamatergic neurons, which is critical for cognitive processes related to MDD, such as negative emotion encoding, reward processing and stress adaptation.^56-58^ Specifically, the LHb plays a key role in regulating reward behaviours^59,60^ through its projections to the dopaminergic ventral tegmental area (VTA) and serotonergic raphe nucleus, where it exerts inhibitory control over monoaminergic activity. Often described as the brain’s ‘anti-reward’ system,^58,59^ the LHb is critical to processing negative reward signals and mediating aversive behavioural responses.

Ketamine’s ability to block NMDAR-dependent bursting activity in the LHb^4^ is thought to contribute to its rapid antidepressant effects, as it temporarily removes the inhibitory brake on the reward system. Ketamine reduces LHb bursting activity and overall firing rates,^61^ which are elevated in depression models,^4,62^ particularly targeting neurons with high basal burst frequencies by blocking NMDARs in their open state, leading to a sustained suppression of LHb activity lasting up to 24 h in mice.^61,63^ Further, evidence suggests that ketamine continues to block NMDARs beyond its half-life, potentially due to its retention within the NMDAR channel pore.^61,64^ This mechanism may explain the prolonged suppression of LHb activity and the sustained antidepressant effects observed following ketamine administration.

### Brain reward circuitry

Depression is frequently accompanied by anhedonia and disrupted reward processing (Box 3), particularly within fronto-striatal regions, which are key components of the brain’s reward circuitry. These circuits, including the nucleus accumbens (NAc), caudate nucleus and PFC, play essential roles in reward processing, motivation and the experience of pleasure. Functional connectivity disruptions in these areas are consistently reported in individuals with MDD.^66,73^

Box 3 Anhedonia Anhedonia is a core symptom of depression, frequently used to assess depressive episodes and inform diagnosis.^65^ It is characterized by deficits in reward processing, encompassing effort, motivation, reward anticipation, learning, pleasure and valuation. Symptoms of anhedonia vary in presentation and complexity among individuals and several neurotransmitters, such as dopamine, GABA, opioids and glutamate, are involved.^66^ The neurobiology of anhedonia involves disruptions in circuits and structures within the reward system, including the ventral tegmental area (VTA), striatum and prefrontal cortex (PFC), though it remains incompletely understood.^67^ Anhedonia is challenging to treat effectively, as conventional antidepressants often fail to address it adequately. Residual symptoms persist, impacting quality of life and increasing relapse risk. Anhedonia is also a key indicator of depression severity, suicidality and treatment outcome.^68^ Currently, there is no FDA-approved treatment specifically targeting anhedonia. Ketamine has shown promise as anti-anhedonic agent,^6,69-72^ although the precise mechanisms underlying this effect remain unclear.

Ketamine has been observed to reduce anhedonia and modulate reward processing across both preclinical and clinical studies.^5,74,75^ Recent evidence suggests that ketamine has both prohedonic and anti-anhedonic effects.^76^ Prohedonic effects refer to increased reward responsiveness in non-anhedonic states (e.g. recreational contexts), while anti-anhedonic effects involve reversing reward deficits induced by chronic stress. In a recent study using the probabilistic reward task^77,78^ in rats, ketamine briefly increased reward responsiveness in non-stressed animals but produced longer-lasting anti-anhedonic effects in chronically stressed rats, persisting for up to a week.^76^ In humans, two exploratory studies^6,7^ suggest that ketamine produces rapid and specific anti-anhedonic effects in TRD, independent of overall mood improvement. These effects were linked to region-specific metabolic changes in reward-related brain areas—including the dorsal anterior cingulate cortex (dACC), putamen, hippocampus and orbitofrontal cortex (OFC). These results suggest that ketamine may specifically target anhedonia through distinct, region-specific neurobiological mechanisms. Consistent with findings in non-human primates,^79^ Morris *et al*.^80^ demonstrated that ketamine normalized subgenual anterior cingulate cortex (sgACC) responses to monetary rewards in humans, as shown by task-based functional MRI (fMRI), and this neural change was associated with reductions in anhedonia. Studies comparing healthy subjects and patients with TRD treated with ketamine suggest that ketamine can normalize brain connectivity patterns associated with depression. Specifically, ketamine appears to enhance functional connectivity between the dorsal and ventral striatum and the PFC, with these changes being particularly pronounced in treatment responders.^81,82^ Notably, these connectivity changes are also strongly correlated with reductions in anhedonia.^81^ Additional evidence indicates that ketamine enhances fronto-striatal connectivity^83^ while reducing connectivity between the default mode network (DMN) and regions such as the sgACC^84^ and the insula,^85^ aligning these connectivity measures more closely with those observed in healthy controls.

Interestingly, ketamine’s impact on reward-related brain circuits may occur independently of measurable clinical improvements in anhedonia. For instance, a recent study involving 37 drug-free, remitted depressed participants found that ketamine reduced dysconnectivity between the ventromedial PFC (vmPFC) and reward-related regions, such as the NAc, shortly after drug administration.^86^ However, these effects appear to be transient. When fronto-striatal connectivity was reassessed ∼10 days after a single ketamine infusion, these changes were no longer evident.^83,85^ This suggests that ketamine’s effects on the reward circuit are transient, and repeated treatments may be necessary to sustain its effect.

Overall, these findings highlight ketamine’s potential to modulate disrupted reward circuits in MDD, offering a promising mechanism for alleviating anhedonia and improving functional outcomes, particularly in individuals with TRD. However, limitations such as small sample sizes, variability in imaging modalities, scan timing, and dosages necessitate cautious interpretation. Standardized protocols and larger studies are warranted to validate and expand upon these findings.

### Negatively biased memories

Animal studies suggest that ketamine’s effects on emotional memories may differ from conventional antidepressants.^8,87^ Using a rodent affective bias model, researchers tested the effect of ketamine and other rapid-acting antidepressants on biases induced during positive or negative affective states.^8,87^ In this model, animals learn to associate specific substrates with a reward (food pellet) under either affective manipulation (e.g. restraint stress) or control conditions. Affective bias is then measured by presenting the two reinforced substrates simultaneously and assessing the animals’ preference.^88^ Although the learned reward value of each substrate is identical, animals typically avoid the stimulus experienced under the negative manipulation (i.e. a negative bias in recall). In this rodent model, when ketamine is given before the preference test (but after the learning phase) this negative bias in memory was attenuated, but there was no effect preference test performance when it was administered during the learning phase.^8^ The opposite effect was seen with conventional antidepressants, such as the serotonin and noradrenaline reuptake inhibitor venlafaxine. These findings suggest that ketamine reduces negative affective bias in encoded memories, whereas venlafaxine is involved in new information processing but is not able to affect previously encoded material.^8^ The different patterns of effect on this affective bias paradigm may provide important insight into the timescale of their clinical action. Unlike venlafaxine, which requires days to weeks of experience to exert its effects, ketamine reduces negative memory recall without necessitating new information processing.

A recent study demonstrated that ketamine selectively attenuates negative affective bias and explored its neural mechanisms of sustained antidepressant effects in animals.^87^ This research proposed a two-phase neuropsychological model: in the first phase, ketamine rapidly modulates medial PFC (mPFC) circuits, reducing negative biases. In the second phase, memories are retrieved and re-encoded with positive valence, a process dependent on protein synthesis in the mPFC and influenced by reward-associated cues reactivated shortly after treatment.^87^

Of note, in the context of negative processing biases, research on ketamine’s effects on emotional processing has produced mixed findings. Some studies have shown that ketamine influences the recognition of specific emotions, such as sadness^89^ (during ketamine administration), fear and happiness (∼20 min post-infusion).^90^ fMRI data from healthy participants indicate that ketamine reduces activation to fearful faces in the amygdala, limbic and visual processing regions, while increasing activation to neutral faces in visual areas—suggesting a blunted neural response to threat and altered salience processing during infusion.^91^ Similarly, Reed *et al*.^92^ reported limited effects on facial emotion recognition accuracy, with patients with MDD showing reduced activity after ketamine infusion that resembled the baseline activity of healthy controls under placebo, suggesting a normalization of function during emotional processing. Notably, variability in administration protocols and experimental designs—many of which were designed to investigate emotional blunting in the context of psychotic symptomatology—limits the generalizability of these findings to mood disorders.

Ketamine’s effects on memory reconsolidation, a process dependent on NMDAR activity and protein synthesis within the mPFC,^93-98^ further highlight its ability to reorganize synaptic architecture and update memories with new information. Memory reconsolidation, a time-dependent process where reactivated stable memories temporarily destabilize and incorporate new content, aligns with ketamine’s impact on affective bias. Further supporting evidence comes from a study on individuals with high alcohol consumption, where ketamine reduced drinking behaviour only when administered after the retrieval of maladaptive alcohol-related memories.^9^ This included reductions in drinking days, alcohol consumption and long-term drinking levels, suggesting ketamine can disrupt appetitive memories in humans. This early evidence intriguingly suggests that ketamine may be able to disrupt maladaptive memories, which may be a key mechanism of antidepressant action; however, the effects of ketamine on affective memory recall in patients with MDD remains to be tested.

Additionally, ketamine administration has been associated with changes in implicit self-associations.^99^ A recent trial investigating whether pairing ketamine with positive self-association training could prolong its antidepressant effects found that combining ketamine with an automated paradigm targeting implicit self-associations extended the duration of ketamine’s antidepressant response.^100^ These findings provide an important foundation for the future combination of rapid-acting antidepressants like ketamine with automated interventions designed to enhance self-related learning.

### Stress resilience

Stress resilience is a complex concept, often described as the capacity to adapt and recover quickly after stress, and maintain normal functioning despite adversity.^101^ Recent preclinical evidence suggests that administering ketamine before an acute stressor can prevent the development of depressive- or post-traumatic stress disorder (PTSD)-like behaviours in animals.^102,103^

Brachman *et al*.^10^ demonstrated that ketamine (30 mg/kg) administered 1 week before chronic social defeat (SD) reduced immobility time in the Forced Swim Test (FST) and increased social interaction in the Dominant Interaction (DI) test, indicating enhanced stress resilience. Of note, ketamine administered after stress (30 mg/kg) did not significantly affect depressive behaviours in the FST, suggesting that its protective effects are most effective when given pre-stress.^10^ Prophylactic ketamine administration increased ΔFosB expression in the ventral dentate gyrus and hippocampus of stressed mice but not control subjects. ΔFosB, a transcription factor regulating synaptic plasticity in brain reward regions, is induced by chronic stress. These findings suggest that prophylactic ketamine may increase stress resilience by modulating neural traces that encode stress experiences in the hippocampus.^104^

A small randomized, placebo-controlled, proof-of-concept study aimed to translate these findings in humans testing whether ketamine could reduce behavioural and physiological effects of an acute stressor when administered 1 week before stress induction in healthy volunteers.^105^ In this study, 24 healthy participants were randomized to receive either ketamine or midazolam before undergoing the Trier Social Stress Test. Although no statistically significant differences in behavioural and biological read-out of stress were observed, ketamine showed a moderate-to-large effect in reducing anxiety (Cohen’s *d* = 0.7) and a significant reduction in salivary α-amylase in the ketamine group, suggesting potential resilience effects that warrant further investigation in larger studies.

Notably, recent studies have also investigated the use of ketamine for preventing postpartum depression (PPD) in humans.^11,106-109^ These studies suggest that administering ketamine during scheduled caesarean sections, before the onset of psychopathology, may help protect against the development of mood and anxiety disorders following childbirth. This would be of great importance considering that PPD affects an estimated 10%–20% of mothers^110,111^ and currently only one medication, brexanolone, is available and received FDA approval for the treatment of PPD.^112^ Although ketamine administration appears helpful in preventing PPD symptoms, further research is needed to confirm its efficacy and safety in preventing PPD among subjects undergoing caesarean section.

## Conclusions

The use of ketamine as an antidepressant has represented a major paradigm shift in the treatment of depression. Its use has demonstrated that rapid-acting medications for MDD are achievable and has highlighted the potential of a novel treatment target, the glutamate system. However, critical questions remain, including the mechanisms behind its rapid and sustained effects, its impact on brain neurocircuitry, and its potential as a pro-resilience agent. Addressing these issues is particularly important given the significant limitations associated with the use of ketamine, including its potential for abuse, and largely unknown long-term safety profile. The knowledge gained thus far should guide the development of new rapid-acting antidepressants with improved safety profiles and set the stage for another promising 25 years of clinical research in the field.
